# Connecting the Dots: Exploring Psychological Network Analysis as a Tool for Analyzing Organizational Survey Data

**DOI:** 10.3389/fpsyg.2022.838093

**Published:** 2022-05-03

**Authors:** Senne Letouche, Bart Wille

**Affiliations:** Department of Developmental, Personality and Social Psychology, Ghent University, Ghent, Belgium

**Keywords:** psychological networks, organizational surveys, employee perceptions, leadership attitudes, innovative work behavior

## Abstract

Organizations allocate considerable resources in surveys aimed at assessing how employees perceive certain job aspects. These perceptions are often modeled as latent constructs (e.g., job satisfaction) measured by multiple indicators. This approach, although useful, has several drawbacks such as a strong reliance on local independence and a lower performance in exploratory contexts with many variables. In this paper, we introduce psychological network analysis (PNA) as a novel method to examine organizational surveys. It is first argued how the network approach allows studying the complex patterns of attitudes, perceptions, and behaviors that make up an organizational survey by modeling them as elements in an interconnected system. Next, two empirical demonstrations are presented showcasing features of this technique using two datasets. The first demonstration relies on original organizational survey data (*N* = 4270) to construct a network of attitudes and behaviors related to innovative work behavior. In the second demonstration, drawing on archival leadership data from an organization (*N* = 337), the focus lies on comparing structural properties of leadership attitude networks between subsamples of supervisors and non-supervisors. We conclude this paper by discussing how PNA constitutes a promising avenue for researching organizational phenomena which typically constitute a set of interconnected elements.

Organizations spend a great deal of time, energy and money on assessing attitudes, behaviors and/or perceptions of their employees (Rogelberg and Stanton, 2007). Organizational surveys are widely used for this purpose, systematically collecting employees’ perceptions about a broad range of psychological constructs including (but not limited to) employee engagement, job satisfaction and turnover intentions. This assessment can then be used to observe trends in the perceptions of employees (Lang et al., 2018), serve as input for future decisions (Nieva, 2003), or design attitude change interventions (Huebner and Zacher, 2021).

Organizational surveys are typically conducted in a similar way: Respondents are presented with a set of items or *indicators* each belonging to one of the overarching psychological constructs of interest. Indicators are statements that refer to behaviors, attitudes, or perceptions. Next, respondents are asked to indicate the degree to which they agree with each statement. Related indicators are subsequently combined into an overall score that represents the construct. By doing so, organizational surveys all share the same underlying assumption: The psychological construct causes the item scores. This is consistent with causal latent variable theory: Items hang together because they are caused by the same underlying latent construct (Costantini et al., 2015a). For example, innovative work behavior (IWB) - a popular construct in organizational research and surveys (Anderson et al., 2014) - is typically measured by combining aspects of idea generation, idea promotion, and idea realization (Janssen, 2000). The scale thus assumes that higher levels of IWB is what *causes* individuals to engage in the generation, promotion, and/or realization of ideas.

Several drawbacks of this approach have been noted. First, grouping items under latent constructs builds on the premise that no direct causal relationships exist between indicators both within and across constructs (Schmittmann et al., 2013). The latent construct accounts for the covariance between indicators. This covariance is assumed to disappear after controlling for the latent construct, also referred to as *local independence* (Schmittmann et al., 2013). Going back to innovation, the common cause (i.e., IWB) accounts for the covariance between idea generation, idea promotion, and idea realization. Similarly, covariance with external variables is entirely attributed to the latent construct since indicators are considered interchangeable. However, meaningful relationships between indicators are likely to exist within and across many organizational phenomena. For instance, research suggests that idea generation often precedes idea realization (Baer, 2012; Škerlavaj et al., 2019), thereby violating the assumption of local independence. Likewise, idea generation and idea realization have been found to be distinct activities, requiring different resources (Škerlavaj et al., 2019) and being differently related to other constructs (e.g., communication; Hülsheger et al., 2009).

Second, conducting surveys can require a considerable amount of time and effort (Kavanaugh et al., 2012). To ensure an efficient use of resources, it is vital to get the most out of a survey. As a result, a *broad range* of employee attitudes, behaviors, and perceptions toward work-related issues is typically assessed. This also makes sense from a theoretical perspective since the relationship between different organizational phenomena is usually influenced by many situational variables (Johns, 2006). However, traditional approaches (e.g., structural equation modeling or SEM) typically perform less well to study such complex interplays with large sets of variables (Hevey, 2018; Letina et al., 2019).

Finally, many employee attitudes and behaviors are dynamically intertwined. Research by Newman et al. (2010) showed substantial overlap between different employee attitudes and bidirectional effects are also observed in many organizational phenomena (e.g., innovation; Anderson et al., 2014). As a result, when assessing attitudes and/or behaviors in surveys, it is often difficult to pinpoint in advance the specific elements that will relate to one another (and those that will not). However, studying large sets of variables in an exploratory manner is precisely where contemporary methods have the tendency to fall short (Epskamp et al., 2017; Ito, 2020) since they are more appropriate in confirmatory situations (Costantini et al., 2015b; Letina et al., 2019).

Building upon recent developments in psychological research methods, this paper examines employee attitudes, perceptions, and behaviors from a psychological network perspective. Rather than interpreting survey elements as a function of a set of underlying latent constructs, psychological network analysis (PNA) conceptualizes items as autonomous entities (Borsboom and Cramer, 2013; Schmittmann et al., 2013). These entities mutually exert causal force on one another, thereby forming a *complex system*. In this regard, PNA helps to circumvent the aforementioned shortcomings by exploratorily analyzing a greater number variables at once (Letina et al., 2019) and by focusing on the interplay between indicators. Additionally, and importantly, the approach also comes with a range of indices that convey information about structural properties of these complex systems (i.e., network centrality and connectivity) that reflect aspects of the phenomenon under study that are both conceptually and empirically distinct from metrics provided by contemporary methods (Costantini et al., 2015a).

This paper makes two contributions to the field of organizational survey research. First and foremost, this is, to the best of our knowledge, the first application of psychological network theory and analysis to a broad range of organizational measures. Building on the work of Carter et al. (2019) and Lowery et al. (2021), who successfully applied PNA to job satisfaction and job performance, respectively, the current paper focuses on a wider set of employee perceptions and attitudes. Thereby, this study answers to the explicit call for studies to examine many job attitudes and their complex interactions (Carter et al., 2019). As we will argue below, exploring the connections between employee attitudes using PNA is a hypothesis generating endeavor with the potential to further our conceptual understanding of organizational phenomena. Second, this paper also makes a practical contribution. Network analysis yields statistical measures (i.e., centrality, connectivity) that traditional methods cannot provide (Hevey, 2018). It is shown how these measures can be used as complementary tools for those working with organizational surveys.

This paper proceeds by first outlining the general ideas behind PNA. Next, the approach is applied to organizational survey data collected in two organizations. In Demonstration 1, the conceptual basis of the network approach is illustrated after which we empirically demonstrate the relevance of network centrality in particular. In demonstration 2, the focus lies on examining structural differences in network properties between subgroups of employees within an organization (i.e., supervisors versus non-supervisors).

## The Network Approach

Networks analysis is a method to analyze the interrelations between elements or *nodes* which are mutually interconnected through *edges* (Bringmann et al., 2019). Importantly, the meaning and nature of these nodes and edges depends on the phenomenon being studied. Perhaps the most common application in the field of organizational psychology is social network analysis (SNA), which models the edges between identifiable nodes such as employees, departments, or organizations. The network structure can subsequently be related to aspects of the phenomenon under study. For instance, prior work has investigated how one’s position in a SNA relates to one’s status (Agneessens and Wittek, 2012), or how edges between nodes facilitate the formation of friendships (Lowery et al., 2021).

The current study focusses on a different application of network analysis called *psychological network analysis* (PNA). PNA conceptualizes constructs and their interrelations as complex systems of interconnected elements. Nodes represent psychological variables that differ across people (e.g., symptoms, attitudes, and/or behaviors). Edges represent the unknown statistical relationship between two nodes and need to be estimated. An important distinction with traditional approaches to study the relationship between these elements is that nodes in PNA are treated as autonomous entities with causal power (Schmittmann et al., 2013; De Schryver et al., 2015). For instance, symptom-level depression networks are a popular application of PNA wherein depression is conceptualized as “a causal interplay of symptoms” (Borsboom and Cramer, 2013, p. 91). Studies have subsequently demonstrated how depression networks emerge and behave as a whole (Bringmann et al., 2019). PNA received rather modest attention so far in the context of organizational behavior, namely for investigating job satisfaction (Carter et al., 2019) and job performance (Lowery et al., 2021). However, it can be argued that such causal interplays are likely to function at the core of many other organizational phenomena and that PNA can therefore be deployed to model the relations between a range of variables often included in organizational surveys.

First, it allows for a better understanding of organizational phenomena by focusing on patterns of relationships between behaviors, perceptions, and attitudes that might go unnoticed when analyzing surveys at the construct level. Consider the following items of the IWB scale: “I generate original solutions for problems” (idea generation), and “I put effort in the development of new things” (idea realization). A network approach treats these items as autonomous entities that hang together for a causal, logical reason (i.e., it might be easier to develop new things if one has previously generated original solutions for problems). Moreover, a network approach to organizational survey data is well-suited to study differential relationships between indicators and one or more variables of interest (e.g., communication). Whereas traditional approaches would establish an overall relationship between IWB and communication, the network approach provides a more fine-grained insight into which specific innovative behaviors and which communication aspects incite each other. Examining how attitudes incite each other, cluster, and/or emerge therefore has the potential of deepen our understanding of organizational survey data.

Second, by focusing on the interrelations between a broader range of variables simultaneously, PNA better enables exploratory research aimed at examining complex systems of patterns of employee attitudes. Hypotheses following these patterns can subsequently be tested in confirmatory settings. For example, there is an extensive line of research which supports the positive impact of job autonomy on job satisfaction (Griffin et al., 2001). However, contextual variables such as job characteristics (e.g., skill variety; Park, 2016) and leadership (e.g., feedback; Dodd and Ganster, 1996) have been shown to influence this relationship. Network analysis allows exploring these unique patterns across and within organizations without the need to make strong assumptions about their direction or causality.

Another final advantage of this approach is that network analysis offers several unique statistical metrics that inform about structural properties of the network. Specifically, the current study explores both network centrality and connectivity characteristics. Centrality refers to the relative importance of a node in a network and has previously been used to study networks in many areas, ranging from transportation (Liu et al., 2019) to gene research (Siddani et al., 2013; Hindumathi et al., 2014). In psychological networks, *central* nodes (i.e., survey elements) have more or stronger connections with other nodes, making them more important for the network than other *peripheral* elements. These structurally important nodes are particularly useful candidates for interventions within organizations since a change in these elements is more likely to affect other elements (Carter et al., 2019). Additionally, network *connectivity* or *density* reflects the degree to which nodes are connected to each other. In the context of attitude networks, densely connected networks are more resistant to change. In the context of organizational surveys, connectivity informs whether changing job attitudes will be more difficult for specific groups (e.g., functional departments, geographical regions, or hierarchical levels). We argue that this approach has the potential to provide organizations with practical tools that allow for future decisions or interventions to be tailored to the specific needs of relevant groups based on topological features of their network structure.

In sum, we argue that PNA offers several features that are complementary to more traditional methods for analyzing organizational survey data. To this end, the paper is built around two empirical demonstrations, in which we illustrate the meaning of (a) centrality in attitude networks (i.e., demonstration 1) and (b) within-organization differences in network structures (i.e., demonstration 2).

## Demonstration 1: Centrality in Attitude Networks

In psychological network theory, centrality refers to the importance of a particular node relative to other nodes in the network and is generally determined by the number of connections. Several centrality indices have been specified with strength centrality being the most common one (Costantini et al., 2015a; Epskamp et al., 2018; Bringmann et al., 2019). Expected influence, an extension of strength centrality that considers both the magnitude and the direction of the connected edges (Robinaugh et al., 2016) is used as the centrality metric in this study. Expected influence is the summed weight of the edges that a node shares with the remaining nodes in the network. Formula 1 presents the mathematical expression of expected influence: a_*ij*_ represents an adjacency matrix with binary elements (1 = edge present, 0 = edge absent) between node i and node j; w_*ij*_ represents an adjacency matrix whose elements range from −1 to 1, indicating the edge weight between node i and node j (Robinaugh et al., 2016).


(1) $${\text{EI}}_{\text{i}}=\int_{\text{j}=0}^{\text{N}}{\text{a}}_{\text{ij}}\,{\text{w}}_{\text{ij}}$$

In terms of interpretation, highly central nodes are more important for determining the dynamics and the structure of the network, and as a result, the psychological phenomenon under investigation (Bringmann et al., 2019). In the context of job attitudes, central nodes in a network of job satisfaction have been shown to be more likely to ‘ripple through’ or affect change in other elements of job satisfaction (Carter et al., 2019). The purpose of this first demonstration is to show how centrality can ameliorate our understanding of a broad network of employee attitudes related to aspects of innovation.

### Method

#### Participants and Procedure

Data was collected in a large Belgian service organization using an online survey conducted June 2021. This demonstration is built around IWB as a focal construct since it lends itself well for PNA. A selection of four additional variables were also included in the analyses given their potential relevance for IWB: job autonomy, communication, team cohesion, and workload. A total of 4,336 employees were instructed to complete the survey, which was done by 4,270 participants. This translates into a response rate of 98%. The average age of participants was 47.76 years (*SD* = 10.00) and 27% identified as male. The average career tenure was 25.64 years (*SD* = 10.86), and the average organizational tenure was 16.13 years (*SD* = 10.86). Ethical approval for this study was granted by the Ethical Committee of Ghent University. Item descriptions of the nodes are detailed in Table 1.

**TABLE 1:** Description of the items and their label.

Element	Item label	Item content
Innovative work behavior	Idea generation	Generating, developing, and communicating ideas
Innovative work behavior	Idea promotion	Mobilizing support for/championing ideas
Innovative work behavior	Idea realization	Transforming innovative ideas into applications
Autonomy	Autonomy process	Autonomy over how the work is done
Autonomy	Autonomy planning	Autonomy over the planning
Autonomy	Autonomy pace	Autonomy over the work pace
Autonomy	Autonomy monitor	Supervisor does not monitor
Team cohesion	Cohesion member	Feeling as a member of the organization
Team cohesion	Cohesion morale	Happy to belong to the organization
Team cohesion	Cohesion belong	Seeing one as part of the organization
Communication	Online formal	Meeting colleagues online formally
Communication	Offline formal	Meeting colleagues face-to-face formally
Communication	Online informal	Meeting colleagues online informally
Communication	Offline informal	Meeting colleagues face-to-face informally
Workload	Workload pace	Having to work fast
Workload	Workload amount	Having to work much
Workload	Workload extra	Having to work extra

#### Measures

##### Job Autonomy

Job autonomy was measured using the four-item scale developed by Janssen (2000). An example item is “I can decide for myself how to get a job done.” The reliability of the scale was adequate (Cronbach’s α = 0.71, McDonald’s ω = 0.71). The response format ranged from 1 = *Strongly disagree* to 7 = *Strongly agree*.

##### Innovative Work Behavior

IWB was measured using the nine items developed by Janssen (2000). This IWB scale foresees three items for each of the three subdimensions: idea generation, idea promotion, and idea realization. Participants were asked to indicate how often they performed a range of innovative work behaviors. The response format ranged from 1 = *Never* to 7 = *Always*. An example of an item for idea generation is “creating new ideas for difficult issues.” Cronbach’s alpha was 0.95, McDonald’s omega was 0.97.

##### Team Cohesion

Team cohesion was measured using the three-item Perceived Cohesion Scale adopted by Chin et al. (1999). An example item is “I see myself as part of this group.” Cronbach’s alpha was 0.84, McDonald’s omega was 0.85.

##### Communication

Following McAlpine (2018), communication was measured by capturing communication differences in the mode (offline vs. online) and content (formal vs. informal). The combination of these two aspects resulted in four items which were presented to participants on a seven-point Likert scale ranging from 1 = *Strongly disagree* to 7 = *Strongly agree*. An example item for online formal communication is “Employees regularly communicate with each other in an online, formal setting (e.g., online meeting).”

##### Workload

In line with Dubbelt et al. (2016), workload was measured using three items that assessed aspects of quantitative demands of the job. A sample item is “I have to work fast” (i.e., work pace). Items are scored on a seven-point Likert-scale, ranging from 1 = *Strongly disagree* to 7 = *Strongly agree*. The Cronbach’s alpha of the items in this sample was 0.91, McDonald’s omega was 0.91.

#### Analysis

Analyses were performed using the open-source R software and the packages ‘bootnet’ (Epskamp et al., 2018), ‘networktools’ (Jones, 2020) and ‘qgraph’ (Epskamp et al., 2012). Missing data was handled using pairwise deletion. The network was estimated using Gaussian Markov random field estimation and implementing graphical LASSO set to 0.5. Edges represent partial correlations (i.e., controlling for the other nodes in the network). The graphical LASSO penalization causes small edges to reduce to exactly zero, resulting in a sparser network (Costantini et al., 2015a). R-code for the analysis is available as Supplementary Material.

Network analysis assumes that nodes represent unique entities. Two nodes that were to measure the same underlying construct would display an inflated correlation. Therefore, IWB items were combined to form a single node for each subscale. Furthermore, the goldbricker function was used to examine if nodes constitute a similar construct or process (Jones, 2020). This function considers two nodes to be indicative of a similar construct or process if they elicit similar correlations with other nodes (less than 25% of divergent correlations, *p* = 0.01). Results indicated that there were no nodes with similar correlation patterns.

### Results

**FIGURE 1:**
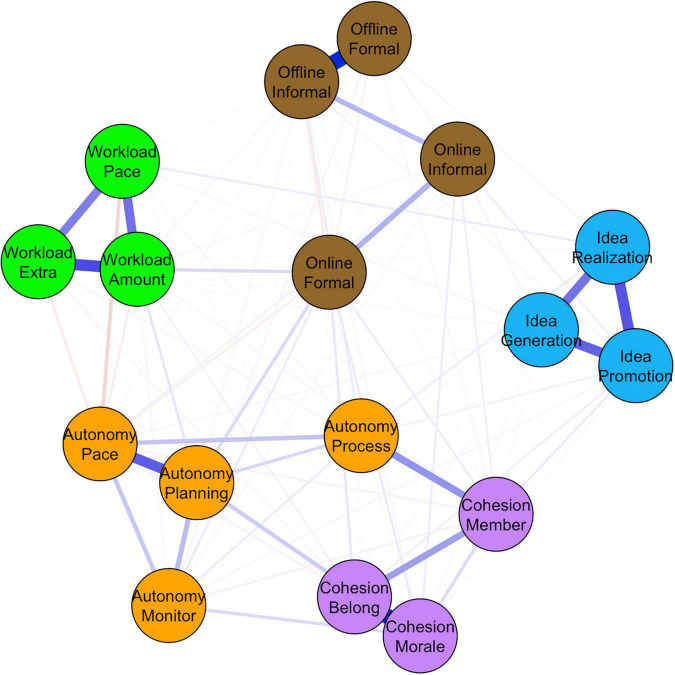
Network of employee perceptions, attitudes, and behaviors related to innovative work behavior. Blue edges represent positive relationships, red edges represent negative relationships. The thickness is proportional to the magnitude of the relationship.

Figure 1 presents the network structure constructed from the organizational survey. Nodes represent single items or scales from the survey and edges represent their statistical relationship (i.e., partial correlations). The thickness of an edge corresponds to the strength of that relationship. For instance, autonomy over how the work is done (*autonomy process*) is more strongly related to feelings of being a member of the organization (*cohesion member*) than to meeting colleagues face-to-face in an informal manner (*offline informal*). The color of edges reflects the sign of the relationship (blue = positive; red = negative).

This visualization immediately illustrates one the benefits of the network approach. Instead of having to look at 91 statistics describing the associations between 17 elements in complex matrices, networks offer an intuitive representation of the same information (Costantini et al., 2015a). Importantly, because of the cross-sectional nature of this dataset, no conclusions can be made regarding the direction of the relationships between nodes. As such, the negative edge between online informal communication (*online informal*) and idea generation (*idea generation*) suggests that higher online informal communication results in less idea generation but generating more ideas could also decrease the amount of informal offline communication. We return to this point in the discussion of the paper.

Once a network has been constructed, its structure provides information on how different elements influence each other on a group-level (i.e., group-level conditional independence; Hevey, 2018). For example, turning innovative ideas into applications (idea realization) hangs together with, among others, generating ideas (idea generation), mobilizing support for those ideas (idea promotion), the pace of work (workload pace) and autonomy over how one’s work is done (autonomy process). In turn, the pace of work negatively relates to autonomy of one’s work pace (autonomy pace), the amount of work (workload extra) and having to work extra (workload extra). This suggests that idea realization occurs in a work environment characterized by limited workload, no hierarchical control, and an emphasis on earlier stages of the innovation process.

Importantly, the main difference with traditional techniques to visualize and/or analyze the relationships between indicators within and across constructs is that the selected indicators now function as unique entities. For instance, traditional techniques would explain the negative edge between autonomy over one’s work pace (*autonomy pace*) and the pace of one’s work (*workload pace*) by relying on the latent constructs *job autonomy* and *workload*. The network approach suggests the latter edge is indicative of the causal, logical explanation that if employees have autonomy over their work pace, they will less likely experience pace workload and vice versa. Thereby, it is not necessarily assumed that autonomy over the planning of one’s work is equally related to the pace of one’s work (as would be the case in latent approaches).

#### Network Accuracy and Stability

As mentioned earlier, psychological networks are constructed on sample data. Therefore, investigating the accuracy of the sample-estimates is required before interpreting structural properties of the network (e.g., centrality, connectivity). Specifically, the following features are examined here: (a) the stability of the centrality indices; (b) the accuracy of the edge weights.

##### Centrality Stability

Centrality stability informs about the accuracy of centrality indices. It expresses how well the order of centralities remains after subsetting the data. Centrality stability is estimated with the CS-coefficient, which represents the maximum proportion of cases that can be dropped, such that with 95% probability the correlation between original centrality indices and centrality of the subsets is 0.70 or higher (Epskamp et al., 2017). Several authors note that this coefficient should exceed the threshold of 0.25 (Glück et al., 2017) and preferably above 0.50 (Hevey, 2018). Following a 1,000-sample bootstrap, the CS-coefficient of the current network was 0.75 for expected influence, which suggests that expected influence is a stable indicator of centrality.

##### Edge-Weight Accuracy

Edge-weight accuracy assesses the accuracy of the connections in the estimated network by calculating confidence intervals (CIs) using non-parametric bootstrapping (i.e., using a 1,000-sample bootstrap, a 95% confidence bootstrapped CI was calculated for the edge weights). If these CIs are large, then it becomes hard to interpret the edge weights. As a result, fewer nodes and/or more observations will result in more reliable edges. The accuracy assessment of the edges shows small to moderate CIs overlap around the edge weight estimates, indicating stable results (i.e., little variability in edge weight estimation in each sample). We refer the reader to Epskamp et al. (2018) for a broader discussion of edge weight accuracy as well as centrality stability.

##### Centrality in Organizations

**FIGURE 2:**
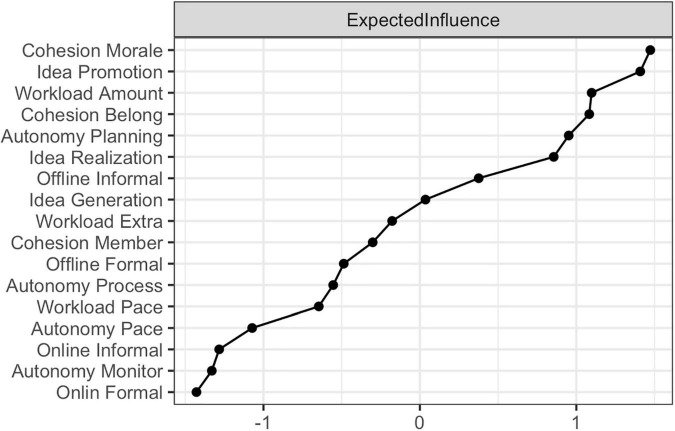
Expected influence centrality of the employee perceptions, attitudes and behaviors related to innovative work behavior. Standardized expected influence centrality of the attitudes and behaviors related to IWB.

The expected influence of the nodes of the organizational survey is plotted in Figure 2. The y-axis displays the nodes, and the x-axis shows the corresponding expected influence as standardized z-values. Being happy to belong to the organization (1.15; *cohesion morale*), mobilizing support for ideas (1.13; *idea promotion*) and the amount of work one must do (1.06; *workload amount*) were the three nodes with the highest expected influence. Conversely, online formal communication with colleagues (*online formal*) was the node with the lowest expected influence. This suggests future interventions aimed at increasing IWB could focus on these central attitudes or behaviors and their interactions. After all, changes in these elements should have more influence than interventions focusing on peripheral nodes.

## Demonstration 2: Connectivity Differences Within Organizations

The results of organizational surveys can also be used to identify possible differences between subgroups within organizations, for instance depending on the department where one works, the kind of job one occupies, or one’s demographic group. However, no research up to date has compared subgroups in organizations on structural properties of psychological networks. We explore how connectivity and centrality of job attitudes might be different across different groups of employees within one organization.

Whereas centrality focuses on single nodes, connectivity or *density* applies to the entire network. Connectivity is calculated by the average correlation between all the nodes in the network. Greater connectivity typically implies the tendency of nodes in the network to be more resistant to change (Dalege et al., 2017). For instance, connectivity differences in attitude networks of two groups reveals which group would be more easily persuaded to change its behavior (Zwicker et al., 2020).

With regard to the factors influencing network connectivity, Carter et al. (2019) used the notion of attitude strength to explain how increased exposure to attitude objects increases connectivity of the attitude network. Specifically, using job tenure as a proxy for exposure, Carter et al. (2019) demonstrated that certain aspects of the job satisfaction network became gradually more dense or strongly connected with higher job tenure. Drawing from this work, we explore whether higher leadership exposure results in more densely connected leadership attitude networks. Holding supervisory responsibilities could facilitate a more strongly connected leadership attitude network because this position is likely to (a) increase the volume of knowledge one has about leadership, (b) heighten awareness of what constitutes ‘good’ or ‘bad’ leadership, and (c) facilitate the accessibility of information about what constitutes leadership. As a result of this increased interaction with the attitude object, dependencies between different leadership behaviors can be expected to become clearer, resulting in a denser network.

For this purpose, we gathered data on perceptions toward leadership of both employees with and without supervisory responsibilities (further referred to as ‘supervisors’ and ‘non-supervisors’) within an organization. All the nodes in our model therefore concern perceptions or attitudes that people hold toward their supervisors in the organization. Specifically, each perception was considered a key leadership behavior that the participating organization identified as important. In addition to the general connectivity of the leadership attitude network, we also explore how centrality of different leadership nodes might differ for supervisors and non-supervisors. Greater experience with leadership responsibilities could lead to a shift in the relative importance of certain leadership aspects, as shown by more or fewer associations with other aspects of leadership. No specific expectations are formulated with respect to the centrality of particular leadership nodes.

### Method

#### Participants and Procedure

This demonstration uses archival survey data collected in 2018 in a sample of employees (*N* = 337; 44% male) from a Belgian department of an international business advisory firm. The survey assessed perceptions of employees toward their leaders. Participants were on average 32.79 years old (*SD* = 9.23). Employees were considered leaders if supervisory responsibilities were part of their job role. Of the 337 employees, 181 were identified as supervisors versus 156 non-supervisors. Missing data was handled using pairwise deletion. All analysis were performed using the R software (R Core Team, 2020) and the packages ‘qgraph’ (Epskamp et al., 2012), ‘bootnet’ (Epskamp et al., 2018), and ‘NetworkComparisonTest’ (van Borkulo et al., 2017). The research was conducted according to the ethical rules presented in the General Ethical Protocol of the Faculty of Ghent University. R-code for the analysis is available as Supplementary Material.

#### Measures

The survey covered ten theoretically distinct leadership attitudes assessing a key leadership behavior that the firm identified as important (see Table 2). Example items include control (“I feel controlled by my supervisor”), and trust (“I can count on my supervisor to be trustworthy”). Employees presented with these items indicated the degree to which their supervisor displayed these behaviors (1 = *Strongly disagree* to 7 = *Strongly agree*).

**TABLE 2:** Description of the items and their label.

Element	Item label	Item description
Open dialogue	Open dialogue	“I can engage in an open conversation with my supervisor if I want to”
Good terms	Good terms	“I have a good relationship with my supervisor”
Trust	Trust	“I can count on my supervisor to be trustworthy”
Support	Support	“I receive adequate support from my supervisor”
Autonomy	Autonomy	“My supervisor entrusts me to work autonomously”
Feedback	Gives feedback	“My supervisor provides me with sufficient feedback”
Feedback acceptance	Receives feedback	“My supervisor is open to my feedback”
Control	Control	“I feel controlled by my supervisor”
Awareness	Awareness	“My supervisor is alert to the atmosphere of group members”
Development opportunities	Development	“My supervisor provides sufficient opportunity to develop myself”

### Results

#### General Network Structures

Similar to Demonstration 1, this network was constructed using partial correlations and graphical LASSO regularization with a tuning parameter set to 0.5. This makes the interpretation of the two group networks identical to the demonstration 1. Stability assessments showed moderate confidence intervals around the edge weights. The CS-coefficients for the networks of non-supervisors and supervisors were 0.36 and 0.52, respectively, which is well-above the 0.25 cut-off (Glück et al., 2017). This indicates that the strength centrality index is rather stable and can be interpreted.

**FIGURE 3:**
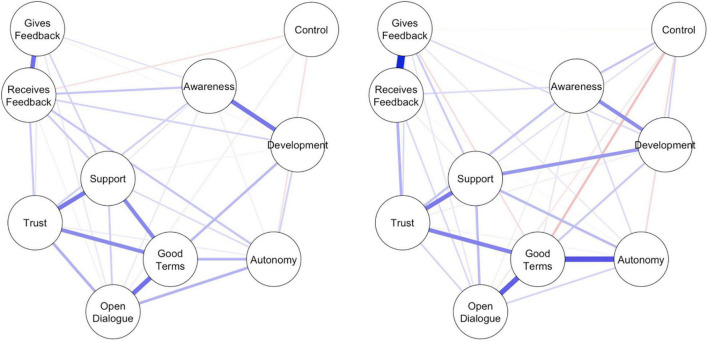
Survey networks of leadership attitudes of both non-supervisors **(Left)** and supervisors **(Right)**. Blue edges represent positive relationships, red edges represent negative relationships. Thicker edges represent stronger associations.

The resulting two networks (see Figure 3) display both similarities and differences. Of note are the strong connections between providing feedback (*gives feedback*) and being open to feedback (*receives feedback*) in both groups. Being aware of the atmosphere (*awareness*) and providing subordinates opportunities to develop (*development*) also display a similar association. However, receiving adequate support from supervisors (*support*) and providing subordinates the opportunity to develop themselves (*development*) is strongly associated for supervisors, but not for non-supervisors. Similarly, a good relationship with one’s supervisor (*good terms*) and receiving adequate support (*support*) were only strongly connected in non-supervisors.

#### Network Comparisons

To test for potential differences between the two networks of leadership attitudes, we used the Network Comparison Test (NCT; van Borkulo et al., 2017). The NCT examines the invariance of three different network aspects: (a) global network structure; (b) edge strength; and (c) global network strength or connectivity.

The first test examines whether the structure of the network as a whole is identical across the two subpopulations based on examining connection strength between edges from subpopulations. It informs about differences in the similarity of edge weight distributions. Results were not significant (*M* = 0.273, *p* = 0.334), indicating that the network structure is identical across the two subpopulations. Because the network structure test yields no significant results, testing group-level differences for specific edges is not recommended as this increases the likelihood of Type 1 errors (van Borkulo et al., 2017).

Second, we compared global network strength or connectivity across the two subpopulations using the NCT, by comparing the absolute sum of all the edges between groups. The test statistic S (i.e., the difference in global strength) was 0.66 (*p* < 0.05), which suggests that the attitude network was denser for supervisors compared to non-supervisors.

Finally, we tested for potential differences in the strength centrality between both networks. Out of the ten nodes in the network, only *the quality of given feedback* (‘Gives Feedback’) differed significantly between the two networks (*p* < 0.01). The amount of given feedback was less central for non-supervisors than for supervisors.

## General Discussion

The management and improvement of positive employee perceptions is vital for organizations. Understanding how employees feel about different aspects of their work environment and how these perceptions influence each other is therefore paramount to science and practice. Organizational surveys are a popular means to achieve this, often assessing a broad range of variables with the goal of initiating change processes to improve organizational effectiveness (e.g., Huebner and Zacher, 2021). Building on recent developments in job attitude research (Carter et al., 2019) and network methodology (van Borkulo et al., 2017; Epskamp et al., 2018), this study is the first to explore a broad range of employee perceptions collected in surveys through the lens of PNA. PNA conceptualizes perceptions, attitudes, and behaviors as autonomous entities in a dynamic system. Items are “part of the construct instead of indicators of the construct” (De Schryver et al., 2015, p. 5), and can exert causal force on other employee attitudes.

In demonstration 1, 17 attitudes and behaviors related to innovation were modeled as a complex system in which they hang together for causal, logical reasons. The resulting network demonstrated good edge-weight accuracy and stable expected influence. *Cohesion morale*, *idea promotion*, and *workload amount* showed the strongest expected influence centrality. This suggests that, relative to all other elements, these three nodes are the most influential employee perceptions in this network of innovation attitudes and behaviors. Changes in these elements cause proportionally greater changes to the rest of the network compared to peripheral attitudes. Although the results of this study need to be interpreted in their organization-specific context, taking into regard the centrality of these organizational survey elements might prove useful for determining starting points for interventions aimed at improving employee attitudes related to IWB.

In addition, demonstration 2 illustrates how potential differences between subgroups within organizations can be examined. Specifically, the Network Comparison Test (NCT) was used to compare leadership attitudes of supervisors and non-supervisors on three network properties: network structure, connectivity, and centrality. The overall structure of the network was found to be identical since the distributions of edge weights did not significantly differ between supervisors and non-supervisors. However, differences did emerge with regard to connectivity and centrality. Consistent with theoretical expectations, the leadership attitudes of supervisors were more densely connected compared to non-supervisors. The two networks thus significantly differed with respect to global strength. This is consistent with the idea that higher exposure to an attitude object –in this case the leadership role– fosters conditional dependencies between leadership attitudes (Carter et al., 2019). Finally, the centrality of the node *gives feedback* differed significantly between the two groups, in the sense that it was more central for supervisors. It could be that supervisors’ experience with leadership results in a higher accessibility of information on what constitutes ‘good’ or ‘bad’ leadership behaviors. This could, in turn, cause that element (i.e., the amount of given feedback) to become more influential. This result would be consistent with traditional research findings on leadership, highlighting that supervisor feedback is crucial for employees (Su et al., 2019).

### Implications

Our findings have several practical and theoretical implications. From a practical point of view, the results of our study provide practitioners with several powerful tools to both examine and –ultimately– improve employee perceptions. Centrality can be applied to examine trends in the relative importance of organizational survey elements. Next, centrality might prove useful for determining starting points for interventions aimed at changing the broader attitudinal network (von Klipstein et al., 2021). In addition, we illustrated how the network approach can also be used to compare different subpopulations within organizations. Specifically, connectivity informs about the ease with which change will occur, whereas differences in centrality inform about the relative importance of nodes across groups. These findings can prove useful for organizations who wish to allocate their resources in the most efficient way (i.e., tailored to the specific needs of groups).

In addition to these practical implications, PNA can also foster theoretical developments. Outside organizational psychology, this approach has already dramatically changed how researchers look at important phenomena such as clinical disorders (e.g., van Borkulo et al., 2015), physical health (e.g., Nudelman et al., 2018) or personality traits (e.g., Costantini et al., 2015b). More recently, Carter et al. (2019) and Lowery et al. (2021) were among the first to argue how network analysis can further our understanding of important organizational constructs such as job satisfaction and performance, respectively. The current study extends these advancements in two ways. First, by considering a broader set of employee attitudes and perceptions, it illustrates how the network approach can also lead to new insights into how different organizational phenomena co-occur or co-develop. Our study shows that improving belonging might boost IWB (and covariates) since changes in this perception create the strongest ripple effect to other perceptions and behaviors. This study also underpins that reinforcing autonomy requires the consideration of unique aspects of autonomy and workload. The pace of work was related to feelings of autonomy over one’s pace but far less with other aspects of autonomy. This finding would go unnoticed when analyzing these variables as latent constructs. Second, when investigating between-group comparisons (i.e., demonstration 2), the current study was also the first to apply PNA to the leadership domain in particular. There is a long tradition of leadership research aimed at differentiating between various styles or behavioral categories, subsequently modeled as (latent) factors. Here, leadership perceptions are studied at a lower level as causal forces mutually influencing each other in a dynamic system. This has the potential to shed new light on leadership in organizations. Specifically, we showed that leaders’ attitude networks showed a higher connectivity, perhaps due to their increased interaction with the focal construct (i.e., leadership). It was also shown that feedback was more influential for supervisors compared to non-supervisors.

### Limitations and Future Research

This study has some limitations. First, the cross-sectional nature does not allow for conclusions about directionality of effects between the different employee perceptions. Nevertheless, both networks reveal group-level conditional independency in complex systems of a great number of variables simultaneously (Hevey, 2018). This exploratory feature leads to the generation of hypotheses of causal dynamics (von Klipstein et al., 2021). For instance, following the results of demonstration 1, future research could examine why formal online meetings are associated with less informal offline communication, but more informal online communication. Holding formal online meetings perhaps installs a norm where people opt for online events more frequently. Future longitudinal studies could investigate how network structures as a whole change over time. Similarly, future research can investigate other organizational phenomena, beyond IWB and leadership, using PNA. To give only one example, the concept of organizational culture is typically defined as a pattern of shared values, attitudes, and thoughts (Schein, 1990) which hang together as an interconnected system or web (Hartnell et al., 2019). Yet, this fundamental principle of interconnections between culture elements has received little attention so far in culture research, and PNA opens the way for new insights in this area as well.

Finally, future research can also study additional network properties in organizational survey data, such as clustering (Hevey, 2018). Clustering is a network feature which has proven useful in the field of clinical psychology, for instance by showing how different PTSD symptoms form communities (De Schryver et al., 2015). In the context of organizations, detecting clusters reveals which job attitudes or behaviors have the tendency to closely relate to each other and thus share information in a sensible way.

## Conclusion

The current study is one of the first to apply psychological network analysis to organizational survey data. By modeling a broad array of employee perceptions, attitudes, and behaviors as an interconnected set of causal forces, this approach allows for a new view on important phenomena within organizations (e.g., innovation, leadership), and how they hang together. Given the burgeoning attention for network approaches in various disciplines of psychology, we foresee a growing number of applications in organizational contexts as well. Future research can build on the current work to further explore the ways in which network analysis can enhance our understanding of people in work settings.

## Data Availability Statement

The raw data supporting the conclusions of this article will be made available by the authors, without undue reservation.

## Ethics Statement

The studies involving human participants were reviewed and approved by the Ethical Committee of the Faculty of Psychology and Educational Sciences of Ghent University. The patients/participants provided their written informed consent to participate in this study.

## Author Contributions

Both authors listed have made a substantial, direct, and intellectual contribution to the work, and approved it for publication.

## Conflict of Interest

The authors declare that the research was conducted in the absence of any commercial or financial relationships that could be construed as a potential conflict of interest.

## Publisher’s Note

All claims expressed in this article are solely those of the authors and do not necessarily represent those of their affiliated organizations, or those of the publisher, the editors and the reviewers. Any product that may be evaluated in this article, or claim that may be made by its manufacturer, is not guaranteed or endorsed by the publisher.

## Funding

This research was funded by the Flemish Agency for Innovation and Entrepreneurship under contract agreement HBC.2019.2621.

## Supplementary Material

The Supplementary Material for this article can be found online at: https://www.frontiersin.org/articles/10.3389/fpsyg.2022.838093/full#supplementary-material
