# Supplementary material for: Connecting the Dots: Exploring Psychological Network Analysis as a Tool for Analyzing Organizational Survey Data
Source: Front Psychol. 2022 May 3;13:838093. doi: 10.3389/fpsyg.2022.838093 (PMC9110883; doi:10.3389/fpsyg.2022.838093)
Supplement: Supplementary file 1 [file Data_Sheet_1.docx]

**Supplementary Materials**

**1. Edge Weights Matrix Figure 1.**

|  | Idea Generation | Idea Promotion | Idea Realization | Autonomy Process | Autonomy Planning | Autonomy Pace | Autonomy Monitor | Cohesion Member | Cohesion Morale | Cohesion Belong | Offline Informal | Offline Formal | Online Informal | Online Formal | Workload Pace | Workload Amount | Workload Extra |
| --- | --- | --- | --- | --- | --- | --- | --- | --- | --- | --- | --- | --- | --- | --- | --- | --- | --- |
| Idea Generation | 0 | 0.42 | 0.37 | 0.01 | 0 | 0.02 | 0.01 | -0.01 | -0.01 | 0 | 0 | 0 | -0.03 | -0.01 | 0.02 | 0.01 | 0.01 |
| Idea Promotion | 0.42 | 0 | 0.46 | 0.03 | 0.01 | 0 | 0.02 | 0.05 | 0.01 | 0.04 | 0.02 | 0 | 0.04 | 0 | 0.01 | 0 | 0.01 |
| Idea Realization | 0.37 | 0.46 | 0 | 0.04 | 0 | 0.01 | 0.01 | 0 | 0 | 0.01 | 0 | 0.02 | 0 | 0 | 0.05 | 0.01 | 0.01 |
| Autonomy Process | 0.01 | 0.03 | 0.04 | 0 | 0.1 | 0.16 | 0.05 | 0.29 | 0.01 | -0.01 | 0.05 | 0 | -0.01 | -0.02 | -0.02 | 0 | 0 |
| Autonomy Planning | 0 | 0.01 | 0 | 0.1 | 0 | 0.44 | 0.18 | 0 | 0.14 | 0.02 | -0.01 | -0.01 | -0.01 | 0.1 | 0 | 0.07 | 0 |
| Autonomy Pace | 0.02 | 0 | 0.01 | 0.16 | 0.44 | 0 | 0.15 | -0.03 | 0.03 | -0.02 | 0.01 | 0.01 | 0.03 | -0.04 | -0.11 | -0.04 | -0.06 |
| Autonomy Monitor | 0.01 | 0.02 | 0.01 | 0.05 | 0.18 | 0.15 | 0 | -0.03 | 0.1 | 0 | -0.02 | -0.03 | -0.02 | 0.05 | 0 | 0.02 | 0 |
| Cohesion Member | -0.01 | 0.05 | 0 | 0.29 | 0 | -0.03 | -0.03 | 0 | 0.1 | 0.26 | 0.01 | 0.02 | 0.04 | 0.05 | 0 | 0.02 | -0.02 |
| Cohesion Morale | -0.01 | 0.01 | 0 | 0.01 | 0.14 | 0.03 | 0.1 | 0.1 | 0 | 0.68 | 0.02 | 0 | 0.05 | 0.05 | -0.01 | 0.01 | -0.01 |
| Cohesion Belong | 0 | 0.04 | 0.01 | -0.01 | 0.02 | -0.02 | 0 | 0.26 | 0.68 | 0 | 0.01 | 0 | 0 | 0.06 | 0 | 0.03 | -0.02 |
| Offline Informal | 0 | 0.02 | 0 | 0.05 | -0.01 | 0.01 | -0.02 | 0.01 | 0.02 | 0.01 | 0 | 0.69 | 0.19 | -0.05 | -0.01 | -0.02 | 0 |
| Offline Formal | 0 | 0 | 0.02 | 0 | -0.01 | 0.01 | -0.03 | 0.02 | 0 | 0 | 0.69 | 0 | 0.03 | -0.03 | 0 | 0 | 0 |
| Online Informal | -0.03 | 0.04 | 0 | -0.01 | -0.01 | 0.03 | -0.02 | 0.04 | 0.05 | 0 | 0.19 | 0.03 | 0 | 0.21 | 0 | -0.02 | 0 |
| Online Formal | -0.01 | 0 | 0 | -0.02 | 0.1 | -0.04 | 0.05 | 0.05 | 0.05 | 0.06 | -0.05 | -0.03 | 0.21 | 0 | 0.01 | 0.1 | 0 |
| Workload Pace | 0.02 | 0.01 | 0.05 | -0.02 | 0 | -0.11 | 0 | 0 | -0.01 | 0 | -0.01 | 0 | 0 | 0.01 | 0 | 0.38 | 0.34 |
| Workload Amount | 0.01 | 0 | 0.01 | 0 | 0.07 | -0.04 | 0.02 | 0.02 | 0.01 | 0.03 | -0.02 | 0 | -0.02 | 0.1 | 0.38 | 0 | 0.49 |
| Workload Extra | 0.01 | 0.01 | 0.01 | 0 | 0 | -0.06 | 0 | -0.02 | -0.01 | -0.02 | 0 | 0 | 0 | 0 | 0.34 | 0.49 | 0 |

**2. Edge Weight Matrices Figure 2 – Non-supervisors**

|  | Open Dialogue | Good Terms | Trust | Support | Autonomy | Gives Feedback | Receives Feedback | Control | Awareness | Development |
| --- | --- | --- | --- | --- | --- | --- | --- | --- | --- | --- |
| Open Dialogue | 0 | 0.33 | 0.18 | 0.11 | 0.18 | 0.05 | 0.03 | -0.04 | 0.05 | 0 |
| Good Terms | 0.33 | 0 | 0.27 | 0.27 | 0.17 | 0 | 0 | 0 | 0 | 0.16 |
| Trust | 0.18 | 0.27 | 0 | 0.31 | 0.05 | 0.06 | 0.12 | 0 | 0.11 | 0 |
| Support | 0.11 | 0.27 | 0.31 | 0 | 0.08 | 0.09 | 0.12 | 0 | 0.05 | 0.03 |
| Autonomy | 0.18 | 0.17 | 0.05 | 0.08 | 0 | 0 | 0.13 | -0.07 | 0.04 | 0.1 |
| Gives Feedback | 0.05 | 0 | 0.06 | 0.09 | 0 | 0 | 0.33 | 0 | 0.07 | 0.02 |
| Receives Feedback | 0.03 | 0 | 0.12 | 0.12 | 0.13 | 0.33 | 0 | -0.07 | 0.13 | 0.1 |
| Control | -0.04 | 0 | 0 | 0 | -0.07 | 0 | -0.07 | 0 | 0.03 | 0 |
| Awareness | 0.05 | 0 | 0.11 | 0.05 | 0.04 | 0.07 | 0.13 | 0.03 | 0 | 0.32 |
| Development | 0 | 0.16 | 0 | 0.03 | 0.1 | 0.02 | 0.1 | 0 | 0.32 | 0 |

**3. Edge Weight Matrices Figure 2 – Supervisors**

|  | Open Dialogue | Good Terms | Trust | Support | Autonomy | Gives Feedback | Receives Feedback | Control | Awareness | Development |
| --- | --- | --- | --- | --- | --- | --- | --- | --- | --- | --- |
| Open Dialogue | 0 | 0.37 | 0.1 | 0.15 | 0.1 | 0.06 | 0.09 | -0.06 | 0.04 | 0 |
| Good Terms | 0.37 | 0 | 0.29 | 0 | 0.4 | -0.08 | 0 | -0.14 | 0.06 | 0.12 |
| Trust | 0.1 | 0.29 | 0 | 0.31 | 0.03 | 0.08 | 0.18 | 0 | 0.14 | 0.04 |
| Support | 0.15 | 0 | 0.31 | 0 | 0.16 | 0.12 | 0.04 | 0.07 | 0 | 0.24 |
| Autonomy | 0.1 | 0.4 | 0.03 | 0.16 | 0 | 0.05 | 0.01 | -0.08 | 0.08 | 0 |
| Gives Feedback | 0.06 | -0.08 | 0.08 | 0.12 | 0.05 | 0 | 0.55 | 0.01 | 0.01 | 0.09 |
| Receives Feedback | 0.09 | 0 | 0.18 | 0.04 | 0.01 | 0.55 | 0 | 0 | 0.09 | 0 |
| Control | -0.06 | -0.14 | 0 | 0.07 | -0.08 | 0.01 | 0 | 0 | 0.13 | 0.1 |
| Awareness | 0.04 | 0.06 | 0.14 | 0 | 0.08 | 0.01 | 0.09 | 0.13 | 0 | 0.26 |
| Development | 0 | 0.12 | 0.04 | 0.24 | 0 | 0.09 | 0 | 0.1 | 0.26 | 0 |

**4. R code**

library(“dplyr”)

library(“psych”)

library(“qgraph”)

library(“bootnet”)

library(“networktools”)

# Select proper columns

Columns <- dataframe %>% select(columns)

# Check node redundancy

goldbricker(data, p = 0.001, method = "hittner2003", threshold = 0.25, corMin = 0.5, progressbar = TRUE)

# Construct network using bootnet

Model <- estimateNetwork(Columns, default = "EBICglasso", tuning = 0.5)

# Edge-weight stability

resboot1 <-bootnet(Model, default = "EBICglasso", nBoots = 1000, nCores = 8)

# Centrality stability

resboot2 <-bootnet(Model,

default = "EBICglasso",

nBoots = 1000,

nCores = 8,

type = c("case"))

plot(resboot2, labels = T, order = "sample")

corStability(resboot2, cor = 0.7)
